# The association between recalled parental rearing behavior and depressiveness: a comparison between 1st immigrants and non-immigrants in the population-based Gutenberg Health Study

**DOI:** 10.1186/s12888-020-02755-1

**Published:** 2020-07-13

**Authors:** Eva M. Klein, Elmar Brähler, Katja Petrowski, Ana N. Tibubos, Mareike Ernst, Jörg Wiltink, Matthias Michal, Philipp S. Wild, Andreas Schulz, Thomas Münzel, Jochem König, Karl Lackner, Norbert Pfeiffer, Manfred E. Beutel

**Affiliations:** 1 Department of Psychosomatic Medicine and Psychotherapy, University Medical Center of the Johannes Gutenberg University Mainz, Langenbeckstraße 1, 55131 Mainz, Germany; 2 Medical Psychology and Sociology, University Medical Center of the Johannes Gutenberg University Mainz, Langenbeckstraße 1, 55131 Mainz, Germany; 3 Preventive Cardiology and Preventive Medicine, Department of Medicine 2, University Medical Center of the Johannes Gutenberg University Mainz, Langenbeckstraße 1, 55131 Mainz, Germany; 4 Center for Thrombosis and Hemostasis (CTH), University Medical Center of the Johannes Gutenberg University, Langenbeckstraße 1, 55131 Mainz, Germany; 5 German Center for Cardiovascular Research (DZHK), partner site Rhine-Main, Mainz, Germany; 6 Center for Cardiology I, University Medical Center of the Johannes Gutenberg University Mainz, Langenbeckstraße 1, 55131 Mainz, Germany; 7 Institute for Medical Biostatistics, Epidemiology and Informatics (IMBEI), University Medical Center of the Johannes Gutenberg University Mainz, Langenbeckstraße 1, 55131 Mainz, Germany; 8 Institute for Clinical Chemistry and Laboratory Medicine, University Medical Center of the Johannes Gutenberg University Mainz, Langenbeckstraße 1, 55131 Mainz, Germany; 9 Department of Ophthalmology, University Medical Center of the Johannes Gutenberg University Mainz, Langenbeckstraße 1, 55131 Mainz, Germany

**Keywords:** Migration, Mental health, Depressiveness, Recalled parental rearing behavior, Gutenberg health study

## Abstract

**Background:**

Studies in immigrant youth have suggested differences in parenting patterns by immigration status. Knowledge of variation in recalled parenting pattern and its distinctive impact on mental health in adult immigrants, however, is limited. Therefore, the purpose of the current study was to investigate similarities and differences in recalled maternal and paternal rearing behavior and its association with depressiveness in adult 1st generation immigrants compared to non-immigrants.

**Methods:**

Seven hundred and forty-three 1st generation immigrants (*M* = 57.4, *SD* = 10.1 years) and 6518 non-immigrants (*M* = 60.3, *SD* = 10.7 years) participated in a population-based study. Regarding countries of origin, the largest subgroups were immigrants from Eastern-Europe, Former-SU, and Arabic-Islamic countries. All participants completed the ultra-short version of The Recalled Parental Rearing Behavior-questionnaire and the PHQ-9 assessing depressiveness. Multiple linear regressions with depressiveness as outcome variable were analyzed separately for each facet of parental rearing behavior adjusting for socio-demographic and migration-related variables.

**Results:**

In addition to differences in depressiveness and socioeconomic status, 1st generation immigrants recalled both their mothers and fathers as more controlling and overprotecting than non-immigrants. Parental emotional warmth was negatively associated with depressiveness across all groups. The relationship between parental control, respectively parental rejection and depressiveness, however, varied in direction and severity between the groups.

**Conclusion:**

The results support the notion that parental warmth is a universal protective factor against depressiveness, whereas the impact of parental control on mental health might be more culturally influenced. Analyses point to the importance of considering the unique contribution of fathers’ rearing behavior on mental health, particularly in immigrant samples.

## Background

The impact of immigration on mental health has been a major area of research within the field of social and transcultural psychiatry [1]. The migratory process is generally regarded as a stressful life event [2]. The direction and strength of the relationship between migration and mental health outcomes, however, has remained an issue of debate. In a previous analysis of data from the population-based Gutenberg Health Study, we found that after adjusting for gender, age and socioeconomic status 1st generation immigrants reported significantly higher prevalence of depression (OR = 1.24), generalized anxiety (OR = 1.38), and suicidal ideation (OR = 1.44) than non-immigrants [3]. Among 1st generation immigrants the prevalence of depression was elevated at 10.1% compared to 2nd generation immigrants (7.3%) and non-immigrants (7.4%). A recent meta-analysis found an aggregate prevalence of depression among immigrants of 15.6% [4]. Reduced mental well-being in immigrant groups has been explained by post-migration factors like psychosocial and economic burdens in the receiving country, perceived discrimination and acculturative stress, and stronger health hazards exposure at work [5–8]. While post-migration stressors undoubtedly account for some portion of higher distress among immigrants, the impact of pre-migration experiences has received scant attention in the research on immigrations’ mental health. Providing a theoretical framework, Spallek et al. [9] have suggested a life course approach acknowledging the importance of the critical phase in early childhood and its impact on subsequent mental health outcomes. In line, recent research has argued that aversive experiences in the pre-migration context like trauma exposure significantly increase the risk for acculturative and psychological distress in the post-migration phase [10, 11]. Yet, empirical studies considering explicitly pre-migration life experiences are still limited [9, 11] and mainly applied to refugee populations conceptualized within a trauma framework [12, 13]. In order to deepen the understanding of the current mental health situation of adult immigrants, however, we need to consider more universal factors of early childhood experiences. For example, a large epidemiology body of literature has confirmed that quality of parenting profoundly shapes later human development and mental well-being [14, 15].

Quality of parenting is reflected in recalled parental rearing behavior which has been identified as an important etiological factor in a vulnerability model of psychopathology [16, 17]. Perceptions of parental rearing have been represented by three principal dimensions: rejection/punishment, control/overprotection and emotional warmth [18]. Parental rejection and punishment is characterized by unaffectionate, hostile and/or dismissive parental behavior towards children. Parental control and overprotection refers to a pattern of tight regulation of children’s activities and feelings related to blaming, constricting, and interfering behavior with performance-oriented expectations. Emotional warmth refers to a supportive, affectionate, and praising child-parent interaction. In line with attachment theory [19], parenting styles characterized by high parental control and punishment have been associated with mental disorders like depression and anxiety disorder in adult life. In contrast, emotional warmth provided by the caregiver has been considered as a protective factor for mental health [20–23]. However, it is important to acknowledge that parents’ rearing beliefs, attitudes, and behaviors are embedded in a certain social-cultural context, pointing to cultural variation in childrearing and its distinctive impact on mental health [24, 25].

Empirical studies have shown that family functioning and parenting styles vary between minority groups and native-born families and have a substantial impact on the development of psychopathology like emotional and behavioral problems among migrant youth [26–28]. Children and adolescents in immigrant families are considered at an increased risk of parenting stress and lower family functioning [29–31] due to structural disadvantages, acculturation stress and intergenerational culture conflicts [32, 33]. The difference in parenting may also be explained by cultural contexts and parents’ socialization goals [31]. While some childrearing concepts may be universal and non-cultural specific like protecting and nurturing the child within a responsive child-parent interaction [24, 34, 35], culture-related patterns and values in parenting have been proposed.

A dominant approach in explaining culture differences in parenting is the distinction between individualism and collectivism which can be conceptualized rather as oscillating levels on one dimension than as two opposite poles [36]. Individualistic-oriented cultures tend to emphasize values like self-fulfillment, autonomy, and independence, whereas more collectivism-oriented cultures stress values such as nurturance, compliance, and interdependence [37]. Accordingly, for example the question to which extent to discipline and control children varies between individualistic and collectivism culture backgrounds. Immigrant parents from more collectivistic cultural backgrounds are more likely to use parenting patterns of control and supervision than individualistic Western majority parents [38–40]. Parental (over) control, however, may not be necessarily associated with rejection or a lack of emotional warmth in collectivistic culture, in which this parental pattern seems to be more normative and thus, without detrimental impact on mental health [34, 40]. For example, parental control was positively correlated with anxiety among adolescents of European and American origin, but negatively associated with anxiety among Asian, Arab or Mexican peers [26, 30].

Further, gender-differentiated associations between maternal and paternal rearing and psychological outcomes are underexplored as most studies consider either parents’ or children’s gender. Gender-related phenomena like differences in behavior and socialization, however, are culturally embedded [41] which affect both the conceptions of the parental role and the perception of the child, how they have been raised and treated [42, 43], and not least mental health outcomes. Previous studies in immigrant samples underscored the importance to analyze maternal and paternal rearing behavior separately due to differences in involvement and cultural conceptualization of parental roles [43–45]. Particularly the role of fatherhood and its cross-cultural variation have been understudied [45, 46].

### The current study

Although research has acknowledged the association between parenting and mental health among immigrant youth and adolescents, to the best of our knowledge no study has explored the effects of similarities and differences in recalled parental rearing behavior on distress in immigrant women and men. Our large data set gives us the unique and novel opportunity to identify patterns of parental behavior in 1st generation immigrants living in Germany (as compared to non-immigrants) and to relate these patterns to mental health. Therefore, the current study seeks to address following research questions:

1. How does recalled parental rearing behavior vary between 1st generation immigrants from different countries and non-immigrants?
2. How is recalled parental rearing behavior associated with depressiveness in these subgroups?
3. Do women and men recalled maternal and paternal rearing behavior differently?

Based on theoretical assumptions and empirical evidence from research in migrant youth, we assumed differences between the groups regarding parental control and overprotection. No differences regarding emotional warmth were expected. It was hypothesized that emotional warmth is a universal protective factor against depressiveness, whereas paternal rejection and punishment is a risk factor for depressiveness. Further, it was assumed that control/overprotection differs in its impact on depressiveness in adulthood. As a systematic understanding of how paternal and maternal rearing behavior contributes to mental health in immigrants is still lacking, gender-related effects are analyzed at an exploratory level considering mothers and fathers separately. All analyses were controlled for socioeconomic status due to its association with immigration status.

## Methods

### Procedure and study sample

The Gutenberg Health Study (GHS) is a population-based, prospective, observational single-center cohort study in the Rhine-Main-Region, Germany [47] (for detailed description of the design and the rationale of the GHS see [48]). Targeted individuals were randomly selected from the local registries of the city of Mainz/ Mainz-Bingen stratified in equal strata for age decades, gender, and residence. Individuals, who gave written informed consent, were included in the study. Persons with insufficient knowledge of German language, or those who reported that they were unable to visit the study center on their own (due to their physical and/or mental condition) were excluded (*n* = 734). Among the excluded participants, *n* = 186 did not participate due to insufficient language skills. At baseline, a total of 15,010 participants were examined between 2007 and 2012. This study is based on the follow-up data with *N* = 8365 participants (*M* = 60.0, *SD* = 10.7 years). Due to different socialization processes and different cultural backgrounds, *n* = 1104 2nd generations immigrants were excluded from analysis. Thus, the analysis of this study based on a sample including *N* = 7261 participants.

Migration and generation status were defined according to the German micro census: Participants who were born abroad and migrated to Germany after 1949 were considered as 1st generation immigrants (*n* = 743). Participants reported their country of origin, respectively those of their mother and father. Due to the small numbers of single countries, three groups of origin were combined according former procedures and geographical, historical and religious characteristics (cf. [49]). The group “Eastern Europe” (*n* = 223) included Poland, Czech Republic, Slovakia, former Czechoslovakia, Slovenia, Rumania, Bulgaria and Hungary. Countries of the Former Soviet Union (Russia, Armenia, Azerbaijan, Belarus, Georgia, Latvia, Kazakhstan, Kirgizstan, Lithuania, Uzbekistan, Moldova, Ukraine, Estonia, Lithuania) were combined in the Group “Former Soviet Union” (*n* = 99). The third group “Arabic- Islamic countries” (*n* = 108) comprised Turkey, Afghanistan, Algeria, Bangladesh, Egypt, Gambia, Guinea-Bissau, Jordan, Indonesia, Iraq, Kuwait, Lebanon, Morocco, Pakistan, Senegal, Syria, and Tunisia. *N* = 313 1st generation immigrants came from other countries. Additionally, information about the time of migration and the primarily spoken language at home were assessed. In the sample, *n* = 6518 non-immigrants were considered as reference group.

The study and its procedure were approved by the ethics committee of the Statutory Physician Board of the State of Rhineland-Palatinate and the local and federal data safety commissioners. All study investigations have been followed the Declaration of Helsinki and principles outlined in recommendations for Good Clinical Practice and Good Epidemiological Practice.

### Measures

Sociodemographic variables included gender, age in years, living with partner (no/yes), employment (no/yes), and income in Euro. Education, profession and income were used to calculate the socioeconomic status (SES) ranging from 3 to 21 [50].

Adult’s perception of paternal and maternal rearing styles and behavior in childhood were measured by the ultra-short screening version of The Recalled Parental Rearing Behavior Questionnaire (Fragebogen zum erinnerten Elterlichen Erziehungsverhalten, FEE, [18, 51]). Twelve items assessed the frequency of certain experiences in childhood on a Likert-Scale from 0 = “no, never” to 3 = “yes, always”. Each item was scored for mothers and fathers. Three independent dimensions were calculated for each parent: 1) Paternal/Maternal Rejection and Punishment (Cronbach α = .75), 2) Paternal/Maternal Control (Cronbach α = .65), and 3) Paternal/Maternal Emotional Warmth (Cronbach α = .82). Following the standardized instruction of the scale, no specific instruction for single-parent or (half-) orphans was provided. The reliable and valid scale showed scalar invariance across gender and age [16, 52]. In addition, the questionnaire has been applied in different countries confirming cross-cultural invariance [53–55].

Depressiveness was assessed with the established Patient Health Questionnaire-9 (PHQ-9; [56]). The scale has been shown to be psychometrically equivalent across immigrant groups [57, 58]. Cronbach alpha in the present study was = .82. Participants rated nine items describing criteria of depression on a Likert-Scale from 0 = “not at all” to 3 = “nearly every day”.

### Statistical analysis

For the characteristics of the study population, variables are presented as frequencies or means with standard deviations. *χ²*-tests, respectively *t*-tests were applied for group comparisons. Addressing the first research question, in the first step all 1st generation immigrants in the current sample were analyzed. In the second step, we considered only the three largest subgroups from different countries of origin (Eastern Europe: *n* = 223, Arabic- Islamic countries: *n* = 108, Former Soviet Union: *n* = 99). Due to statistical feasibility and content-related considerations, the next largest immigrants groups were not analyzed in depth (West- and Central Europe: *n* = 93, South Europe: *n* = 69). This applied also for even smaller and more diverse immigrants groups (e.g. South-America: *n* = 13). Multiple linear regressions with depressiveness as outcome variable were analyzed separately for each subscale of the recalled parental rearing behavior questionnaire adjusting for socio-demographic and migration-related variables. Both paternal and maternal parenting variables were included. *P-value* < .05 were considered as worthwhile for further interpretation. Due to the large sample, the exactly reported *p*-values should be interpreted descriptively. Hence, effect sizes (Cohen’s d) were calculated. Statistical analyses were performed using R version 3.5.1.

## Results

Table 1 gives an overview of sociodemographic variables, mental health and recalled parental rearing behavior comparing 1st generation immigrants and non-immigrants. As Table 1 shows, 7261 participants were in included with *n* = 743 of the sample being 1st generation immigrants. 1st generation immigrants were more likely women and younger. They had a lower socioeconomic status and were more often unemployed than Germans without migration background. There was a significant difference in the mental health status: 1st generation immigrants reported to feel more often depressed. Further, 1st generation immigrants differed from non-immigrants with respect to recalled parental rearing behavior: 1st generation immigrants recalled their mothers both as more controlling and overprotecting and as more rejecting and punishing. There was no overall difference regarding maternal emotional warmth. Regarding their fathers, 1st generation immigrants reported more paternal control/overprotection and paternal emotional warmth compared to the reference group. No significant difference emerged on the paternal rejection and punishment scale.

**Table 1:** Demographic characteristics, mental health and recalled parental rearing behavior: 1st generation immigrants vs. non-immigrants in the Gutenberg Health Study

	1st generation Immigrants (*n*=743)	Non-Immigrants (*n*=6,518)	*χ²/t*	*p/d*
***Sociodemographic***				
Gender (women)	52.9 %	48.5 %	5.1	**.022**
Age	57.4 (10.1)	60.3 (10.7)	7.5	**< .0001**
SES	12.31 (4.25)	12.73 (4.48)	2.3	**.021;*d*=.09**
Unemployment (yes)	3.9 %	1.0 %	40.2	**< .0001**
Partnership (yes)	86.3 %	85.9 %	.04	.83
***Mental heath***				
Depressiveness PHQ-9	5.38 (4.12)	4.23 (3.57)	-6.9	**< .0001;*d*=.32**
***Parental rearing behavior***				
Maternal Rejection & Punishment	0.33 (0.50)	0.28 (0.45)	-.2.3	**.020;*d*=.11**
Maternal Control & Overprotection	0.73 (0.65)	0.55 (0.56)	-6.6	**< .0001;*d*=.32**
Maternal Emotional Warmth	1.34 (0.75)	1.34 (0.70)	-.04	.97
Paternal Rejection & Punishment	0.30 (0.47)	0.29 (0.46)	-.28	.77
Paternal Control & Overprotection	0.58 (0.59)	0.42 (0.50)	-.6.2	**< .0001;*d*=.31**
Paternal Emotional Warmth	1.06 (0.75)	0.98 (0.72)	-.2.5	**.0099;*d*=.11**

*Note:* Data are presented in means and standard deviations in brackets or in frequencies; *d* = Cohen’s d

Among 1st generation immigrants, the largest group were immigrants from Eastern Europe. They were in average 25 years old when they immigrated to Germany. The majority of immigrants from Eastern Europe (85%) speaks German at home. The second largest group were participants who were born in Arabic Islamic countries. At the time of migration, they were about 20 years old. Approximatively half of them stated that German is the preferred spoken language at home. In the group of immigrants from the Former Soviet Union 58% reported speaking German at home. While they have been living almost 10 years less in Germany compared to the other immigrant groups, they were comparatively older at the time of migration (*M* = 34 years).

Table 2 compares sociodemographic variables, mental health and recalled parental rearing behavior between 1st generation immigrants from Eastern Europe, Former Soviet Union and Arabic-Islamic countries with non-immigrants as reference group. Whereas no difference between immigrants from Eastern Europe and non-immigrants regarding sociodemographic variables was found, immigrants from Former Soviet Union and Arabic-Islamic countries were younger, more often unemployed and had a lower socioeconomic status than non-immigrants.

**Table 2:** Comparing 1st generation immigrants from Eastern Europe, Former Soviet Union and Arabic- Islamic countries with non-immigrants

	Immigrants from Eastern Europe (*n*=223)	* Test statistics*	Immigrants from Former Soviet Union (*n*=99)	* Test statistics*	Immigrants from Arabic-Islamic countries (*n*=108)	*Test statistics*	Non-Immigrants (*n*=6,518)
***Sociodemographic***							
Gender (women)	53.4 %	*ns*	59.6 %	**4.41; .036**	37.0 %	**5.09; .024**	48.5 %
Age	59.2 (10.8)	*ns*	54.9 (9.3)	**5.72; <.0001;*d*=.50**	53.0 (8.8)	**8.50; <.0001;*d*=.68**	60.3 (10.7)
SES	12.56 (4.17)	*ns*	11.83 (3.97)	**2.19; .030;*d*=.20**	11.53 (4.77)	**12.73; .015;*d*=.27**	12.73 (4.48)
Unemployment (yes)	1.3 %	*ns*	8.1 %	**37.2; <.0001**	5.6 %	**16.2; <.0001**	1.0 %
Partnership (yes)	88.9 %	*ns*	85.9 %	*ns*	82.8 %	*ns*	85.9 %
***Migration-related variables***							
Years living in Germany	33.91 (12.64)	-	21.04 (7.72)	-	32.84 (9.61)	-	
Speaking German at home (yes)	85.5 %	-	58.4 %	-	52.9 %	-	
***Mental health***							
Depressiveness (PHQ-9)	5.42 (3.84)	**-4.44; <.0001; *d*=.33**	5.96 (4.11)	**4.02; .000;*d*=.48**	6.58 (5.27)	**-4.06; .000;*d*=.65**	4.23 (3.57)
***Parental rearing behavior***							
Maternal Rejection & Punishment	0.35 (0.48)	**-2.05; .041; *d*=.15**	0.35 (0.51)	*ns*	0.24 (0.51)	*ns*	0.28 (0.45)
Maternal Control & Overprotection	0.69 (0.61)	**-3.16; .002; *d*=.25**	0.77 (0.72)	**2.73; .008;*d*=.39**	0.82 (0.75)	**-3.20; .002;*d*=.48**	0.55 (0.56)
Maternal Emotional Warmth	1.35 (0.69)	*ns*	1.30 (0.77)	*ns*	1.19 (0.73)	*ns*	1.34 (0.70)
Paternal Rejection & Punishment	0.35 (0.49)	*ns*	0.23 (0.41)	*ns*	0.16 (0.37)	**3.01; .004;*d*=.28**	0.29 (0.46)
Paternal Control & Overprotection	0.56 (0.57)	**-3.21; .002;*d*=.28**	0.54 (0.52)	**2.03; .046;*d*=.24**	0.71 (0.73)	**-3.38; .001;*d*=.58**	0.42 (0.50)
Paternal Emotional Warmth	1.02 (0.71)	*ns*	1.14 (0.77)	*ns*	1.08 (0.75)	*ns*	0.98 (0.72)

Note: presented are χ²/t and p-values; *d* = Cohen’s d; *ns*= not significant

Irrespective of the country of origin, immigrants reported significantly higher levels of depressiveness than Germans without migration background. Further, they consistently recalled both their mothers and fathers as more controlling and overprotecting compared to non-immigrants. Additionally, differences were found between immigrants from Eastern Europe and non-immigrants in maternal rejection with higher levels reported by participants born in Eastern Europe. Immigrants from Arabic-Islamic countries showed the lowest mean scores on the paternal rejection and punishment scale.

The analyses of gender differences in recalled parental rearing behavior within the three subgroups were explorative due to the small sample sizes (Table S1 in supplement). The results showed that women from Eastern Europe and Former Soviet Union remembered their mothers as more rejecting and punishing than their male counterparts. Women from Arabic-Islamic countries reported higher levels of paternal overprotection and paternal emotional warmth than men from Arabic-Islamic countries.

In order to investigate whether the impact of parental rearing behavior on depressiveness varies across the groups, multiple regression analyses were conducted. For this purpose, separate regression models stratified by the subscales of recalled parental rearing behavior were performed with depressiveness as outcome variable (Table 3). Sociodemographic variables were included in the models as control variables. The regression models were also adjusted for migration-related factors (speaking German at home) and country of origin. A dummy variable for the item “speaking German at home” with the category “does not apply” for non-immigrants was created and included in the model. Main effects of maternal and paternal rearing behavior were explored. The specific influence of parental rearing behavior in each group (Eastern Europe, Former Soviet Union, Arabic-Islamic countries; non-immigrants as reference group) were considered in the regression models by adding interaction terms of country of origin and maternal, respectively paternal rearing behavior. Results of the regression models revealed that being a women, younger age, lower SES and not having a partner were risk factors for depressiveness. Across all models, paternal and maternal rejection and punishment as well as paternal and maternal control and overprotection were significantly associated with higher depressiveness, whereas paternal and maternal emotional warmth was negatively associated with depressiveness. Looking at each model more closely, the results suggested that the direction of the relationship between paternal rearing behavior and depressiveness differed between the groups: The results obtained from the regression model considering maternal and paternal control/ overprotection suggested that maternal control/ overprotection was related with higher scores of depressiveness among immigrants from Arabic-Islamic countries. However, paternal controlling and overprotecting behavior seems to be a protective factor for depressiveness in immigrants from Former SU. Paternal rejection/punishment was associated with higher levels of depressiveness among immigrants from Arabic-Islamic countries, the same direction like for non-immigrants. In contrast, for immigrants from Eastern Europe paternal rejection/punishment had an opposite impact on depressiveness: Recalled experiences with a more rejecting and punishing fathers was associated with lower levels of depressiveness. In the regression model with parental emotional warmth as independent variable, no interaction between parental emotional warmth and country of origin emerged. In this model, not speaking German at home was correlated with depressiveness.

**Table 3:** Associations of parental rearing behavior with depressiveness stratified for parental control & overprotection, rejection & punishment and emotional warmth as independent variables using linear regressions

Depressiveness			
**Parental Control (C) & Overprotection (OP)**	*b*	*t*	*p*
*R*^*2*^=.09; *F*(17,6043)=33.8; *p*< .0001			
Age	-.43	-9.65	**< .0001**
Gender (women)	.86	9.60	**< .0001**
SES	-.08	-7.82	**< .0001**
Partnership (yes)	-.64	-4.76	**< .0001**
Eastern Europe	1.1	2.63	**.01**
Arabic-Islamic countries	.80	1.23	.22
Former Soviet Union	1.9	2.94	**.003**
Not Speaking German at home (yes)	.70	1.70	.09
Maternal Control & Overprotection	.78	7.13	**< .0001**
Paternal Control & Overprotection	.42	3.38	**.001**
Maternal C&OP in Eastern Europe	.28	.45	.65
Maternal C&OP in Arabic-Islamic countries	2.4	3.19	**.001**
Maternal C&OP in Former Soviet Union	.96	1.07	.29
Paternal C&OP in Eastern Europe	-.73	-1.11	.27
Paternal C&OP in Arabic-Islamic countries	-1.4	-1.90	.06
Paternal C&OP in Former Soviet Union	-3.1	-2.63	**.01**
**Parental Rejection (R) & Punishment (P)**	*b*	*t*	*p*
*R*^*2*^=.09; *F*(17,6109)=37.0; *p*< .0001			
Age	-.45	-10.3	**< .0001**
Gender (women)	.91	10.20	**< .0001**
SES	-.06	-6.33	**< .0001**
Partnership (yes)	-.76	-5.70	**< .0001**
Eastern Europe	1.2	3.30	**.001**
Arabic-Islamic countries	1.1	2.26	**.02**
Former Soviet Union	1.3	2.58	**.01**
Not Speaking German at home (yes)	.63	1.65	.10
Maternal Rejection & Punishment	.80	6.71	**< .0001**
Paternal Rejection & Punishment	.92	7.94	**< .0001**
Maternal R&P in Eastern Europe	.27	.45	.65
Maternal R&P in Arabic-Islamic countries	-1.0	-1.22	.22
Maternal R&P in Former Soviet Union	-.59	-.51	.60
Paternal R&P in Eastern Europe	-1.2	-2.04	**.04**
Paternal R&P in Arabic-Islamic countries	4.4	3.57	**.0004**
Paternal R&P in Former Soviet Union	.31	0.25	.80
**Parental Emotional Warmth (EW)**	*b*	*t*	*p*
*R*^*2*^=.07; *F*(17,5904)=27.1; *p*< .0001			
Age	-.51	-11.2	**< .0001**
Gender (women)	.98	10.40	**< .0001**
SES	-.07	-6.93	**< .0001**
Partnership (yes)	-.72	-5.23	**< .0001**
Eastern Europe	.81	1.33	.18
Arabic-Islamic countries	1.1	1.34	.18
Former Soviet Union	1.1	1.18	.24
Not Speaking German at home (yes)	.99	2.41	**.02**
Maternal Emotional Warmth	-.30	-3.54	**.0004**
Paternal Emotional Warmth	-.37	-4.41	**< .0001**
Maternal EW in Eastern Europe	-.38	-.85	.40
Maternal EW in Arabic-Islamic countries	1.1	1.38	.17
Maternal EW in Former Soviet Union	-.89	-1.02	.31
Paternal EW in Eastern Europe	.65	1.49	.14
Paternal EW in Arabic-Islamic countries	-.73	-.88	.38
Paternal EW in Former Soviet Union	.90	1.06	.29

*Note: C&OP* Control & Overprotection, *R&P* Rejection & Punishment, *EW* Emotional Warmth; dummy variable (speaking German at home: does not apply) is not presented because not significant

## Discussion

Despite the theoretical notion and empirical research on adolescents from immigrant families that childrearing differs across socio-cultural contexts [27, 28], knowledge of variation in recalled parenting pattern and its distinctive impact on mental health in adult immigrants is limited. In line with etiological models of vulnerability, the findings of this population-based study affirmed that recalled rearing behavior was associated with depressiveness in adults across all groups. Nonetheless, the results provide evidence for differences between 1st generation immigrants from different countries of origin and non-immigrants in their memories of parents’ childrearing pattern and how these childhood experiences affected their adult mental health.

Regarding our first research question and in line with our hypothesis, 1st generation immigrants consistently remembered both their mothers and fathers as more controlling and overprotective compared to non-immigrants. Consistent evidence indicates that higher levels of control over children’s behavior and demanded obedience are more common in collectivist-oriented cultures (e.g. Turkey, Russia) emphasizing interdependence compared to more individualist-oriented cultures emphasizing independence [59]. Parental control in collectivist-oriented cultures aims to teach children to inhibit the expression of their own needs in order to attend to the needs of their cultural in-group maintaining group harmony [40]. Differences in parental control were found both between immigrant parents compared to Western majority parents [26, 30] as well as in cross-cultures studies [40, 60]. Variations in parental control and overprotection are undoubtedly influenced by cultural norms and values. An alternative explanation for the association between immigration status and parental control might be a changing rearing pattern due to the experience of migration. Research suggested that immigrant parents impose higher control and overprotection over their children resulting from their own feelings of anxiety and insecurity as they raise their children in a cultural environment which might be dissimilar from the one in which they grew up themselves [36, 39]. Yet, in our sample most participants had immigrated as adults, having been raised in the same cultural context as their parents. Regarding gender differences, no differences in parental control between women and men were found. This result contrasted those from a recent meta-analysis indicating slightly higher parental control over boys than over girls but with a negligible effect size after controlling for the samples’ ethnicity and socioeconomic status [61].

Compared to participants born in Germany, 1st generation immigrants recalled their mothers slightly more rejecting and punishing. Particularly women from Former SU and Eastern Europe reported the highest levels of maternal rejection and punishment. Hence, the findings of the current study do not support previous research suggesting general harsh and restrictive childrearing behavior among immigrant parents from the Former SU [62], but point to a gender effect. Although we found no overall difference in parental rejection and punishment between 1st generation immigrants and non-immigrants, the analyses considering countries of origin revealed that participants in the Arabic-Islamic group reported the lowest levels of parental rejection and punishment. This finding contrasts the notion of fathers’ traditional disciplinarian role in Arabic-Islamic context [44, 63]. In the same vein, the exploratory analyses of gender differences suggested that particularly women from an Arabic-Islamic country reported the highest levels of parental emotional warmth.

Whereas 1st generation immigrants in the sample did not differ in their remembrance of maternal emotional warmth compared to non-immigrants, they recalled their fathers as emotionally warmer. However, no difference in paternal warmth was identified across the three groups. The findings confirm our hypothesis that an affectionate, supportive and sensitive responsive parental behavior seems to be a universal childrearing concept [24, 45, 64].

With respect to our second research question, our findings suggest that the relationship of emotional warmth and depressiveness is similar between the groups, whereas paternal control, respectively paternal rejection showed a different pattern of relatedness to mental health among the groups. Regarding their mental health status, 1st generation immigrants felt consistently more depressed than non-immigrants with immigrants from Arabic-Islamic countries reporting the highest level of depressiveness across the groups ([3] for detailed discussion of this finding). After controlling for socioeconomic status, which varies between the groups, depressiveness was associated both with parental rejection and punishment and with parental control and overprotection, and negatively associated with parental emotional warmth. In line with attachment theory [65], emotional warmth seems to be protective for mental health and functioning irrespective of immigration status and culture. Numerous studies across different nations and ethnic minority groups have shown that a warm, carrying and accepting child-parent interaction is associated with psychological adjustment (see [66] for a meta-analysis). In contrast, parental control and rejection can result in the internalization of a dysfunctional inner working models of the self and others [67] and induce a feeling of helplessness [68]. Consequently, the individual’s perceived mastery of coping with emotional stress exposure is decreased, elevating the risk for depressiveness in adulthood [16, 69]. However, the findings suggest that meaning and consequences of parental control may be more prone for cultural variance [60]. Parental control reflects a more normative childrearing script in collectivist-oriented cultures and does not necessarily harm individuals’ mental health [70, 71].

Our findings suggest differences in the meaning and consequences of recalled fathers’ rearing behavior. Immigrants from Former SU, who recalled their fathers as controlling and overprotecting, reported less depressiveness. Descriptively, in all immigrant groups paternal control and overprotection was negatively associated with depressiveness compared to non-immigrants, whereas maternal control was positively related to depressiveness across all groups. In accordance, Dwairy and Achoui [72] found that fathers’ rather than mothers’ control had an impact on psychological disorders among adolescent in the west, but not in the east. A possible explanation might be that fathers’, but not mothers’ controlling behavior is experienced as involvement fostering the child-father relationship. The positive impact of parental involvement on the child’s well-being has been demonstrated in immigrant families [73, 74]. The finding that a rejecting father seems to have a particularly negative impact on the mental health of immigrants from Arabic-Islamic countries underscores the unique contribution of fathers. Somewhat counterintuitive was the correlation between paternal rejection and depressiveness among immigrants from Eastern Europe. Although speculative, this result might be explained by an interaction effect with the participants’ gender as in previous studies a strict and authoritarian father reduced the risk for externalizing symptoms, but only for males [14, 44]. To disentangle the complex relationship between parents’ and children’s gender, culture and mental health further research in immigrant samples is required. Nonetheless, the findings of the current study yielded evidence for the assumption that the roles of fathers might differ from the roles of mothers across cultures and minority groups [72], highlighting the importance to consider perceived fathers rearing behavior in future research in immigrants [74, 75].

Despite the explorative nature of the study, the findings have implications for clinical practice in both primary care and transcultural psychotherapy. First, this study underscores the importance to broaden the perspective in the clinical assessment and treatment of common health problems with immigrants by including consideration of early childhood experiences like recalled paternal childrearing styles beyond the exploration of the migration process itself and post-migration experiences of the person. This knowledge enables to evaluate adequately the potential impact of each different phase of migration trajectory on diagnostics, etiology, and pathogeneses of mental health problems among immigrants [76, 77]. Second, current data highlight the importance of acknowledging different normative cultural scripts in childrearing, also those of fathers, which might challenge our assumption which childrearing practices and patterns are universal [24]. Hence, in the clinical context an open and curious stance of the clinicians is crucial to avoid ethnocentric perspectives on parenting.

The current study adds to the existing literature which is primarily based on immigrant youth by studying the complex relationship between immigration, mental health and parenting in adults in a population-based study. However, several limitations of the current study are worth mentioning and might be considered in future research. First, the research design was limited to retrospective self-reports of parental rearing behavior facing the issue of recall bias. Retrospective assessment raises the questions whether parenting experiences are an etiology factor for depression or can be explained by a negative bias of perception in depressed individuals [78]. Yet, empirical research gave evidence for stability of recalled parental behavior across mood changes and over time [79, 80]. The results should be interpreted as subjective mental representations and less as actual experiences of parental rearing during childhood, reflecting a subjective truth which is in line with therapeutic approaches. Despite the notion that parenting has an important impact on mental health in adulthood, the explained variances of depressiveness in the current study is modest which is in line with former studies in adults’ samples [15].

A further limitation is related to a selection bias in our sample which presents a general methodological challenge of surveying populations of immigrant origin [81]: Only immigrants with sufficient language skills were able to participate in the study. As language proficiency and social participation such as the willingness to participate in the current study are key indicators for integration [82], it can be assumed that more separated and marginalized immigrants are underrepresented in the sample examined. From research on acculturation attitudes and parenting behavior it is known that immigrated mothers who are more integrated in the culture of the receiving society tend to adapt their child rearing practices to attitudes and behavior of their non-immigrated counterparts [83, 84]. Hence, immigrated parents oriented strongly towards traditional child rearing values of their country of origin might be excluded in the study leading to potential underestimation of differences in parenting pattern.

Second, despite the strength of the study to consider the unique contribution of mothers and fathers separately, the study is limited by the lack of additional information on characteristics of the family structure in which the child has grown up. In many cultures, caregivers other than parents are involved in childrearing shaping child development [85]. For example, the applied scale did not explicitly measure single-parenting or orphanhood. Hence, we neither considered single parenting nor having more children which both tend to result in higher levels of stress with potential impact on parenting [86, 87]. This is particular important considering that immigrant mothers have on average slightly more children than native German mothers [88], but lower income.

Even though the sampling was based on a random procedure, which is recommend particularly in cross-cultural research [89], a third limitation refers to the combination of immigrants from different countries of origin within one subgroup which was due to the small number of cases from single countries. Therefore, the interpretation and generalizability of our results are limited, although we applied a standard operationalization of migration status according to German micro census. The immigrant population within the subgroups compared in the present study are heterogeneous in terms of cultural identify, language, social situation and acculturation style. The heterogeneity of people and the within-culture variation in one group should be bear in mind while interpreting the results [36]. In the same vein, the recalled rearing practices of the German population without migrations background, which served as reference group, should not be interpreted as “normative” benchmark, particularly when considering the historical German background [52]. Hence, the current findings cannot be generalized uncritically to other socio-cultural contexts and immigrant groups [24, 34]. In line, the current results cannot be applied to 2nd generation migration as rearing patters may change over immigrated generations influenced by different acculturation and socializations processes [90]. In order to develop a full picture of the relationship between migration, childrearing behavior and mental health, additional studies with 2nd generation migration will be needed.

## Conclusion

The results of the current study suggest variation in recalled parenting pattern and its distinctive association with mental health among adult immigrants in Germany. Parental warmth seems to be a universal protective factor against depressiveness, whereas the impact of parental control on mental health might be more likely culturally influenced. The current findings point to the unique contribution of fathers’ rearing behavior on mental health, particularly in immigrant samples. Maternal and paternal roles might differ across cultures and minority groups.

## Supplementary information

**Additional file 1: Table S1.** Gender differences within immigrants from Eastern Europe, Former Soviet Union and Arabic-Islamic countries.

## Data Availability

For approved reasons, some access restrictions apply to the data underlying these findings. Data sets contain identifying participant information, which is not suitable for public deposition. Access to the local database is available upon request to the corresponding author.

## Abbreviations

GHS: Gutenberg Health Study
PHQ: Patient Health Questionnaire-9

## References

[1] Butler M, Warfa N, Khatib Y, Bhui K (2015). Migration and common mental disorder: an improvement in mental health over time?. Int Rev Psychiatry.

[2] Kizilhan JI, Machleidt W, Heinz A (2018). Psychologie der Migration. Praxis der Interkulturellen Psychiatrie und Psychotherapie (Zweite Ausgabe): Elsevier.

[3] Beutel ME, Jünger C, Klein EM, Wild P, Lackner KJ, Blettner M (2016). Depression, anxiety and suicidal ideation among 1 st and 2 nd generation migrants-results from the Gutenberg health study. BMC Psychiatry.

[4] Foo SQ, Tam WW, Ho CS, Tran BX, Nguyen LH, McIntyre RS (2018). Prevalence of depression among migrants: a systematic review and meta-analysis. Int J Environ Res Public Health.

[5] Bermejo I, Mayninger E, Kriston L, Härter M (2010). Psychische Störungen bei Menschen mit Migrationshintergrund im Vergleich zur deutschen Allgemeinbevölkerung. Psychiatr Prax.

[6] de Wit MA, Tuinebreijer WC, Dekker J, Beekman A-JT, Gorissen WH, Schrier AC (2008). Depressive and anxiety disorders in different ethnic groups. Soc Psychiatry Psychiatr Epidemiol.

[7] Missinne S, Bracke P (2012). Depressive symptoms among immigrants and ethnic minorities: a population based study in 23 European countries. Soc Psychiatry Psychiatr Epidemiol.

[8] Pham KTH, Nguyen LH, Vuong Q-H, Ho M-T, Vuong T-T, Vu GT (2019). Health inequality between migrant and non-migrant workers in an industrial zone of Vietnam. Int J Environ Res Public Health.

[9] Spallek J, Zeeb H, Razum O (2011). What do we have to know from migrants’ past exposures to understand their health status? A life course approach. Emerging Themes in Epidemiology.

[10] Li M (2016). Pre-migration trauma and post-migration stressors for Asian and Latino American immigrants: transnational stress proliferation. Soc Indic Res.

[11] Li M, Anderson JG (2016). Pre-migration trauma exposure and psychological distress for Asian American immigrants: linking the pre-and post-migration contexts. J Immigr Minor Health.

[12] Schweitzer R, Melville F, Steel Z, Lacherez P (2006). Trauma, post-migration living difficulties, and social support as predictors of psychological adjustment in resettled Sudanese refugees. Aust N Z J Psychiatry.

[13] Bogic M, Njoku A, Priebe S (2015). Long-term mental health of war-refugees: a systematic literature review. BMC Int Health Hum Rights.

[14] Enns M, Cox B, Clara I (2002). Parental bonding and adult psychopathology: results from the US National Comorbidity Survey. Psychol Med.

[15] Overbeek G, ten Have M, Vollebergh W, de Graaf R (2007). Parental lack of care and overprotection. Soc Psychiatry Psychiatr Epidemiol.

[16] Petrowski K, Brähler E, Zenger M (2014). The relationship of parental rearing behavior and resilience as well as psychological symptoms in a representative sample. Health Qual Life Outcomes.

[17] Rikhye K, Tyrka AR, Kelly MM, Gagne GG, Mello AF, Mello MF (2008). Interplay between childhood maltreatment, parental bonding, and gender effects: impact on quality of life. Child Abuse Negl.

[18] Perris C, Jacobsson L, Linndström H, von Knorring L, Perris H (1980). Development of a new inventory for assessing memories of parental rearing behaviour. Acta Psychiatr Scand.

[19] Bowlby J (1973). Attachment and loss, vol. II: Separation: Basic Books New York.

[20] Ihle W, Jahnke D, Heerwagen A, Neuperdt C (2005). Depression, Angst und Essstörungssymptomatik und erinnertes elterliches Erziehungsverhalten. Kindheit und Entwicklung.

[21] Someya T, Kitamura H, Uehara T, Sakado K, Kaiya H, Tang SW (2000). Panic disorder and perceived parental rearing behavior investigated by the Japanese version of the EMBU scale. Depression Anxiety.

[22] Turgeon L, O'connor K, Marchand A, Freeston M (2002). Recollections of parent–child relationships in patients with obsessive-compulsive disorder and panic disorder with agoraphobia. Acta Psychiatr Scand.

[23] Valiente C, Romero N, Hervas G, Espinosa R (2014). Evaluative beliefs as mediators of the relationship between parental bonding and symptoms of paranoia and depression. Psychiatry Res.

[24] Bornstein MH (2013). Cultural approaches to parenting. Parent Sci Pract.

[25] Darling N, Steinberg L (1993). Parenting style as context: an integrative model. Psychol Bull.

[26] Mousavi SE, Low WY, Hashim AH (2016). Perceived parenting styles and cultural influences in adolescent’s anxiety: a cross-cultural comparison. J Child Fam Stud.

[27] Kouider EB, Koglin U, Petermann F (2015). Emotional and behavioral problems in migrant children and adolescents in American countries: a systematic review. J Immigr Minor Health.

[28] Kouider EB, Koglin U, Petermann F (2014). Emotional and behavioral problems in migrant children and adolescents in Europe: a systematic review. European Child & Adolescent Psychiatry.

[29] Flink IJ, Jansen PW, Beirens TM, Tiemeier H, van IJzendoorn MH, Jaddoe VW (2012). Differences in problem behaviour among ethnic minority and majority preschoolers in the Netherlands and the role of family functioning and parenting factors as mediators: the generation R study. BMC Public Health.

[30] Luis TM, Varela RE, Moore KW (2008). Parenting practices and childhood anxiety reporting in Mexican, Mexican American, and European American families. J Anxiety Disorders.

[31] Lansford JE, Deater-Deckard K, Dodge KA, Bates JE, Pettit GS (2004). Ethnic differences in the link between physical discipline and later adolescent externalizing behaviors. J Child Psychol Psychiatry.

[32] Nomaguchi K, House AN (2013). Racial-ethnic disparities in maternal parenting stress: the role of structural disadvantages and parenting values. J Health Soc Behav.

[33] Dettlaff AJ, Earner I, Phillips SD (2009). Latino children of immigrants in the child welfare system: prevalence, characteristics, and risk. Child Youth Serv Rev.

[34] Bornstein MH (2013). Parenting and child mental health: a cross-cultural perspective. World Psychiatry.

[35] Ekmekci H, Yavuz-Muren HM, Emmen RA, Mesman J, van IJzendoorn MH, Yagmurlu B (2015). Professionals’ and mothers’ beliefs about maternal sensitivity across cultures: toward effective interventions in multicultural societies. J Child Fam Stud.

[36] Prevoo MJ, Tamis-LeMonda CS (2017). Parenting and globalization in western countries: explaining differences in parent–child interactions. Curr Opin Psychol.

[37] Triandis HC (1989). The self and social behavior in differing cultural contexts. Psychol Rev.

[38] Nauck B, Lotter V (2015). Parenting styles and perceived instrumentality of schooling in native, Turkish, and Vietnamese families in Germany. Z Erzieh.

[39] Daglar M, Melhuish E, Barnes J (2011). Parenting and preschool child behaviour among Turkish immigrant, migrant and non-migrant families. Eur J Dev Psychol.

[40] Rudy D, Grusec JE (2006). Authoritarian parenting in individualist and collectivist groups: associations with maternal emotion and cognition and children's self-esteem. J Fam Psychol.

[41] Segall MH, Lonner WJ, Berry JW (1998). Cross-cultural psychology as a scholarly discipline: on the flowering of culture in behavioral research. Am Psychol.

[42] Best DL, Williams JE, Berry JW, Segall MH, Kagitcibasi C (1997). Sex, gender, and culture. Handbook of cross-cultural psychology: Social behavior and applications. Needham Heights.

[43] Nguyen H, Rawana JS, Flora DB (2011). Risk and protective predictors of trajectories of depressive symptoms among adolescents from immigrant backgrounds. J Youth Adolescence.

[44] Lahlah E, Van der Knaap LM, Bogaerts S, Lens KM (2014). Ethnic differences in the effect of perceived parenting on juvenile violent delinquency of Dutch and Moroccan-Dutch boys. J Child Fam Stud.

[45] Miconi D, Moscardino U, Ronconi L, Altoè G (2017). Perceived parenting, self-esteem, and depressive symptoms in immigrant and non-immigrant adolescents in Italy: a multigroup path analysis. J Child Fam Stud.

[46] McKelley RA, Rochlen AB, Wong YJ, Wester SR (2016). Furthering fathering: What we know and what we need to know. APA Handbook of Men and Masculinities.

[47] Wild P, Zeller T, Beutel M, Blettner M, Dugi K, Lackner K (2012). Die Gutenberg Gesundheitsstudie. Bundesgesundheitsblatt-Gesundheitsforschung-Gesundheitsschutz..

[48] Hohn R, Kottler U, Peto T, Blettner M, Munzel T, Blankenberg S (2015). The ophthalmic branch of the Gutenberg health study: study design, cohort profile and self-reported diseases. PLoS One.

[49] Brettschneider A-K, Hölling H, Schlack R, Ellert U (2015). Mental health in adolescents in Germany: a comparison with regard to migration background and country of origin. Bundesgesundheitsblatt, Gesundheitsforschung, Gesundheitsschutz.

[50] Lampert T, Kroll L, Müters S, Stolzenberg H (2013). Measurement of socioeconomic status in the German health interview and examination survey for adults (DEGS1). Bundesgesundheitsbl Gesundheitsforsch Gesundheitsschutz.

[51] Schumacher J, Eisemann M, Brähler E (1999). Rückblick auf die Eltern: Der Fragebogen zum erinnerten elterlichen Erziehungsverhalten (FEE). Diagnostica..

[52] Petrowski K, Paul S, Zenger M, Brähler E (2012). An ultra-short screening version of the recalled parental rearing behavior questionnaire (FEE-US) and its factor structure in a representative German sample. BMC Med Res Methodol.

[53] Deković M, ten Have M, Vollebergh WA, Pels T, Oosterwegel A, Wissink IB (2006). The cross-cultural equivalence of parental rearing measure: EMBU-C. Eur J Psychol Assess.

[54] Arrindell W, Akkerman A, Bagés N, Feldman L, Caballo VE, Oei TP (2005). The short-EMBU in Australia, Spain, and Venezuela. Eur J Psychol Assess.

[55] Arrindell W, Richter J, Eisemann M, Gärling T, Rydén O, Hansson S (2001). The short-EMBU* in East-Germany and Sweden: a cross-national factorial validity extension. Scand J Psychol.

[56] Kocalevent RD, Hinz A, Brähler E. Standardization of the depression screener patient health questionnaire (PHQ-9) in the general population. Gen Hosp Psychiatry 2013;35(5):551–5. Epub 2013/05/15. 10.1016/j.genhosppsych.2013.04.006 23664569

[57] Tibubos AN, Beutel ME, Schulz A, Klein EM, Brähler E, Michal M (2018). Is assessment of depression equivalent for migrants of different cultural backgrounds? Results from the German population-based Gutenberg health study (GHS). Depression Anxiety..

[58] Tibubos AN, Kröger H. A cross-cultural comparison of the Ultrabrief mental health screeners PHQ-4 and SF-12 in Germany. Psychol Assess. 2020;32(7):690–7. 10.1037/pas0000814 32191078

[59] Matsumoto D, Yoo SH, Fontaine J (2008). Mapping expressive differences around the world: the relationship between emotional display rules and individualism versus collectivism. J Cross-Cult Psychol.

[60] Deater-Deckard K, Lansford JE, Malone PS, Alampay LP, Sorbring E, Bacchini D (2011). The association between parental warmth and control in thirteen cultural groups. J Fam Psychol.

[61] Endendijk JJ, Groeneveld MG, Bakermans-Kranenburg MJ, Mesman J (2016). Gender-differentiated parenting revisited: meta-analysis reveals very few differences in parental control of boys and girls. PLoS One.

[62] Shor R (2000). Jewish immigrant parents from the former Soviet Union: a method for studying their views of how to respond to children’s misbehavior. Child Abuse Negl.

[63] Renzaho A, McCabe M, Sainsbury W (2011). Parenting, role reversals and the preservation of cultural values among Arabic speaking migrant families in Melbourne. Australia Int J Intercultural Relations.

[64] Mesman J, Van IJzendoorn M, Behrens K, Carbonell OA, Cárcamo R, Cohen-Paraira I (2016). Is the ideal mother a sensitive mother? Beliefs about early childhood parenting in mothers across the globe. Int J Behav Dev.

[65] Bowlby J (1980). Attachment and loss: loss, sadness and depression (Vol. 3).

[66] Rohner RP, Khaleque A (2010). Testing central postulates of parental acceptance-rejection theory (PARTheory): a meta-analysis of cross-cultural studies. J Fam Theory Rev.

[67] Otani K, Suzuki A, Matsumoto Y, Enokido M, Shirata T (2016). Effects of perceived affectionless control parenting on working models of the self and other. Psychiatry Res.

[68] Garber J, Flynn C (2001). Predictors of depressive cognitions in young adolescents. Cogn Ther Res.

[69] Beutel ME, Tibubos AN, Klein EM, Schmutzer G, Reiner I, Kocalevent R-D (2017). Childhood adversities and distress-the role of resilience in a representative sample. PLoS One.

[70] Dwairy M, Achoui M, Abouserie R, Farah A (2006). Parenting styles, individuation, and mental health of Arab adolescents: a third cross-regional research study. J Cross-Cult Psychol.

[71] Kagitcibasi C (2005). Autonomy and relatedness in cultural context: implications for self and family. J Cross-Cult Psychol.

[72] Dwairy M, Achoui M (2010). Parental control: a second cross-cultural research on parenting and psychological adjustment of children. J Child Fam Stud.

[73] Leyendecker B (2016). Engagement türkischstämmiger Väter im Familien-und Erziehungsalltag fördert das subjektive Wohlbefinden von Kindern/Involvement of Turkish Immigrant Fathers Elevates Children’s Well-Being. Prax Kinderpsychol Kinderpsychiatr.

[74] Leyendecker B, Cabrera N, Lembcke H, Willard J, Kohl K, Spiegler O (2018). Parenting in a new land. Eur Psychol.

[75] Strier R, Roer-Strier D. Fatherhood in the context of immigration. In: Lamb ME, editor. The role of the father in child development. New Jersey: Wiley; 2010. p. 435–58.

[76] Kirmayer LJ, Narasiah L, Munoz M, Rashid M, Ryder AG, Guzder J (2011). Common mental health problems in immigrants and refugees: general approach in primary care. CMAJ..

[77] Graef-Calliess IT, Behrens K, Machleidt W, Heinz A (2018). Kultursensible Diagnostik und migrationsspezifische Anamnese. Praxis der Interkulturellen Psychiatrie und Psychotherapie (Zweite Ausgabe): Elsevier.

[78] Gerlsma C, Mosterman I, Buwalda S, Emmelkamp PM (1992). Mood and memories of parental rearing styles: a comparison of mood effects on questionnaire-cued and free recall of autobiographical memories. J Psychopathol Behav Assess.

[79] Wilhelm K, Niven H, Parker G, Hadzi-Pavlovic D (2005). The stability of the parental bonding instrument over a 20-year period. Psychol Med.

[80] Richter J, Eisemann M (2001). Stability of memories of parental rearing among psychiatric inpatients: a replication based on EMBU subscales. Psychopathology..

[81] Mendez M, Font J (2014). Surveying ethnic minorities and immigrant populations: methodological challenges and research strategies: Amsterdam University press.

[82] Martin S, Maehler D, Behr D, Pötzschke S. Methodische Grundlagen der quantitativen Migrationsforschung: Methoden der Migrationsforschung. Wiesbaden: Springer; 2016. p. 17–59.

[83] Yagmurlu B, Sanson A (2009). Acculturation and parenting among Turkish mothers in Australia. J Cross-Cult Psychol.

[84] Durgel ES, Leyendecker B, Yagmurlu B, Harwood R (2009). Sociocultural influences on German and Turkish immigrant mothers’ long-term socialization goals. J Cross-Cult Psychol.

[85] Bornstein MH, Sawyer J, McCartney K, Phillips D (2006). Family systems. Blackwell Handbook of Early Childhood Development.

[86] Liang LA, Berger U, Brand C. Psychosocial factors associated with symptoms of depression, anxiety and stress among single mothers with young children: A population-based study. J Affective Disorders. 2019;242:255–64. 10.1016/j.jad.2018.08.013 30218920

[87] Östberg M, Hagekull B (2000). A structural modeling approach to the understanding of parenting stress. J Clin Child Psychol.

[88] Pötzsch O (2018). Aktueller Geburtsanstieg und seine Potenziale. Statistisches Bundesamt (Destatis).

[89] Berry JW, Berry JW, Poortinga YH, Segall MH, Dasen PR (2002). Cross-cultural psychology: research and applications: Cambridge University press.

[90] Driscoll AK, Russell ST, Crockett LJ (2008). Parenting styles and youth well-being across immigrant generations. J Fam Issues.

