# Supplementary material for: The association between recalled parental rearing behavior and depressiveness: a comparison between 1st immigrants and non-immigrants in the population-based Gutenberg Health Study
Source: BMC Psychiatry. 2020 Jul 13;20:367. doi: 10.1186/s12888-020-02755-1 (PMC7358206; doi:10.1186/s12888-020-02755-1)
Supplement: Supplementary file 1 — Additional file 1: Table S1. Gender differences within immigrants from Eastern Europe, Former Soviet Union and Arabic-Islamic countries. [file 12888_2020_2755_MOESM1_ESM.docx]

Table S1 (Supplement): Sex differences within immigrants from Eastern Europe, Former Soviet Union and Arabic-Islamic countries

|  | Immigrants from Eastern Europe | | |  | Immigrants from Former Soviet Union (n=99) | | |  | Immigrants from Arabic-Islamic countries (n=108) | | |
| --- | --- | --- | --- | --- | --- | --- | --- | --- | --- | --- | --- |
|  | Women (n=95-115) | Men  (n=82-99) | *t;p* |  | Women (n=38-46) | Men (n=29-37) | *t;p* |  | Women  (n=23-32) | Men  (n=47-52) | *t;p* |
| ***Parental rearing behavior*** |  |  |  |  |  |  |  |  |  |  |  |
| Maternal Rejection & Punishment | 0.42 (0.55) | 0.27 (0.37) | **-2.39; .017;**  ***d*=.32** |  | 0.49 (0.60) | 0.18 (0.30) | **.3.04; .0033; *d*=.63** |  | 0.32 (0.53) | 0.20 (0.50) | *ns* |
| Maternal Control & Overprotection | 0.73 (0.66) | 0.64 (0.55) | *ns* |  | 0.74 (0.76) | 0.81 (0.67) | *ns* |  | 0.93 (0.77) | 0.77 (0.74) | *ns* |
| Maternal Emotional Warmth | 1.29 (0.76) | 1.42 (0.61) | *ns* |  | 1.28 (0.77) | 1.33 (0.65) | *ns* |  | 1.23 (0.74) | 1.17 (0.73) | *ns* |
|  |  |  |  |  |  |  |  |  |  |  |  |
| Paternal Rejection & Punishment | 0.32 (0.50) | 0.38 (0.50) | *ns* |  | 0.26 (0.49) | 0.19 (0.27) | *ns* |  | 0.18 (0.45) | 0.15 (0.33) | *ns* |
| Paternal Control & Overprotection | 0.55 (0.62) | 0.57 (0.52) | *ns* |  | 0.46 (0.49) | 0.65 (0.54) | *ns* |  | 0.91 (0.75) | 0.61 (0.71) | *ns* |
| Paternal Emotional Warmth | 1.05 (0.75) | 0.98 (0.66) | *ns* |  | 1.16 (0.89) | 1.12 (0.59) | *ns* |  | 1.33 (0.76) | 0.95 (0.71) | **-.2.09; .042**  ***d*=.52** |
|  |  |  |  |  |  |  |  |  |  |  |  |
| Depressiveness (PHQ-9) | 5.89 (3.71) | 4.86 (3.93) | *ns* |  | 6.56 (4.63) | 5.05 (3.01) | *ns* |  | 8.07 (6.10) | 5.66 (4.51) | *ns* |

*Note:* *ns*= not significant
